# The Association Between Skeletal Muscle Mass and Surgical Site Infection and Prognosis in Patients Undergoing Free Flap Reconstructive Surgery for Oral Squamous Cell Carcinoma: A Single-Center, Retrospective Study

**DOI:** 10.3390/cancers17101729

**Published:** 2025-05-21

**Authors:** Atsuro Noguchi, Kenji Yamagata, Satoshi Fukuzawa, Kaoru Sasaki, Shohei Takaoka, Fumihiko Uchida, Naomi Ishibashi-Kanno, Mitsuru Sekido, Hiroki Bukawa

**Affiliations:** 1 Department of Oral and Maxillofacial Surgery, University of Tsukuba Hospital, Tsukuba 305-8576, Japan; aturo.19940904@gmail.com (A.N.); sfkuzawa3104@yahoo.co.jp (S.F.); sho.tmdu@gmail.com (S.T.); f-uchida@md.tsukuba.ac.jp (F.U.); nkanno@md.tsukuba.ac.jp (N.I.-K.); hiroki.bukawa@gmail.com (H.B.); 2 Department of Oral and Maxillofacial Surgery, Institute of Medicine, University of Tsukuba, Tsukuba 305-8575, Japan; 3 Department of Plastic and Reconstructive Surgery, Institute of Medicine, University of Tsukuba, Tsukuba 305-8575, Japan; kaoru-ssk@md.tsukuba.ac.jp (K.S.); msekido@md.tsukuba.ac.jp (M.S.)

**Keywords:** oral squamous cell carcinoma (OSSC), free flap reconstructive surgery (FFRS), skeletal muscle mass index (SMI), overall survival (OS), surgical site infection (SSI), extra nodal extension (ENE)

## Abstract

Background/Objectives: Local and systemic factors, including nutritional status, influence the prognosis of oral squamous cell carcinoma (OSCC). Skeletal muscle mass (SMM) loss is a poor prognostic factor in older patients and those with cancer. Herein, we examined the SMM index (SMI), rates of surgical site infection (SSI), and prognosis of 92 patients (59 males and 33 females) who underwent resection and free flap reconstructive surgery (FFRS) between 2013 and 2021. Methods: Preoperative computed tomography was performed to measure SMM at L3. The median SMI was 45.94 and 38.03 cm^2^/m^2^ in males and females, respectively. Patients were classified into low and high SMI groups based on median SMI, and overall survival (OS) was analyzed. Results: Overall, 47 (51.1%) and 45 (48.9%) patients had low and high SMIs, respectively. SSI occurred in 11 (12.0%) patients; wound dehiscence and delayed wound healing were observed in 22 (23.9%). SSI rates were not significantly different between the low and high SMI groups. Conversely, OS was significantly associated with age, pathological N (pN), extranodal extension (ENE), and SMI (high, 81.1%; low, 60.2%). Univariate analyses revealed significant associations between OS and age (≥65 vs. <65 years), SMI (low vs. high), pN (present vs. none), ENE (present vs. none), and albumin (<4.0 vs. ≥4.0 mg/dL). Cox multivariate analysis included SMI (low vs. high; hazard ratio [HR]: 2.339, 95% confidence interval [CI]: 1.008–5.429; *p* = 0.015) and ENE (present vs. none; HR: 7.727, 95% CI: 3.083–19.368; *p* < 0.001). Conclusions: SMI and ENE were identified as independent predictive factors of OS in patients with OSCC undergoing FFRS.

## Simple Summary

Skeletal muscle mass (SMM) loss is a poor prognostic factor in older patients and those with cancer. We determined the SMM index (SMI), rates of surgical site infection (SSI), and prognosis of 92 patients (59 males and 33 females) with oral squamous cell carcinoma (OSCC) who underwent resection and free flap reconstructive surgery (FFRS) from 2013 to 2021. Preoperative computed tomography measured SMM at L3. Patients were classified into low (*n* = 47, 51.1%) and high SMI (*n* = 45, 48.9%) groups by median. SSI occurred in 11 (12.0%) patients, and wound dehiscence and delayed wound healing occurred in 22 (23.9%). Rates of SSI did not differ significantly between the low and high SMI groups. Cox multivariate analysis included SMI (low vs. high; hazard ratio [HR]: 2.339, 95% confidence interval [CI]: 1.008–5.429; *p* = 0.015) and extranodal extension (ENE) (present vs. none; HR: 7.727, 95% CI: 3.083–19.368; *p* < 0.001). SMI and ENE were identified as independent predictive factors of overall survival in patients undergoing FFRS for OSCC.

## 1. Introduction

Local factors, such as clinical cancer stage, and systemic factors, such as nutritional status, influence the prognosis of oral squamous cell carcinoma (OSCC). Loss of skeletal muscle mass (SMM) has been identified as a poor prognostic factor not only in older patients but also in patients with cancer. Moreover, an association between the SMM index (SMI) and OSCC prognosis has been reported [1,2,3]. Free flap complications, including surgical site infection (SSI), have been shown to be linked to SMI [4,5,6]. Although some reports have explored these factors in OSCC, evidence-based reports are currently unavailable. Therefore, we investigated the association between SMI and SSI in the prognosis of patients who had undergone resection and free flap reconstructive surgery (FFRS) for OSCC and reviewed previous reports. The objective of the current study was to clarify the association between SMI and the prognosis of OSCC.

## 2. Materials and Methods

### 2.1. Study Design and Sample

This retrospective cohort study included patients undergoing resection and FFRS between 2013 and 2021 at the Department of Oral and Maxillofacial Surgery, University of Tsukuba Hospital (Ibaraki, Japan). Inclusion criteria were patients with OSCC undergoing their first surgery with FFRS, without preoperative radiotherapy or chemotherapy, and availability of complete imaging data, including abdominal enhancement computed tomography (CT). Exclusion criteria were as follows: recurrent lesions, non-squamous cell carcinoma (SCC) histological diagnosis, follow-up of less than 36 months, and incomplete medical records.

**Figure 1:**
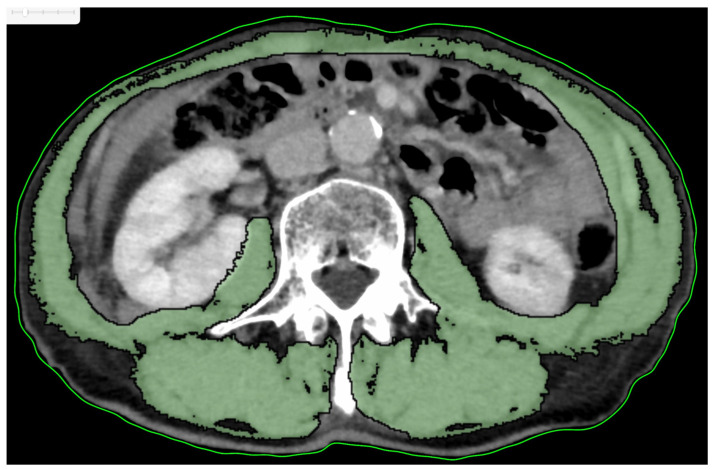
The third lumbar vertebra (L3) was selected as the standard landmark, and the extension from L3 to the iliac crest was selected to measure the muscle cross-sectional area. Skeletal muscle was identified and quantified using Hounsfield unit (HU) thresholds (−29 to +150).

To measure muscle mass, we performed a secondary analysis of electronically stored CT images, which had been used for diagnostic purposes. The third lumbar vertebra (L3) was selected as the standard landmark, and the extension from L3 to the iliac crest was selected to measure the muscle cross-sectional area (CSA). Skeletal muscle was identified and quantified using Hounsfield unit (HU) thresholds (−29 to +150). The L3 region comprises the psoas, paraspinal muscles (erector spinae and quadratus lumborum), and abdominal wall muscles (transversus abdominis, external and internal obliques, and rectus abdominis). Images were analyzed using OsiriX Lite (Pixmeo, Bernex, Switzerland) (https://www.osirix-viewer.com/osirix/patients/, accessed on 7 May 2024). The CSA (cm^2^) of the sum of all of the muscles was computed for each image (Figure 1). Given that this value is linearly related to whole-body muscle mass, it was normalized for stature (L3 SMI, cm^2^/m^2^).

Surgery was the predominant initial treatment for patients with resectable and operable general conditions, along with six cycles of preoperative chemoradiotherapy. According to postoperative pathological results, the high-risk group (presence of extranodal extension (ENE), lymph node (LN) counts ≥2, and a close or positive margin) underwent adjuvant radiotherapy (60~66 Gy) and/or chemotherapy (cisplatin 100 mg/m^2^, 2 or 3 cycles).

For the primary tumor, pathological diagnosis was based on histological grading. Based on the pathological results, postoperative therapy was performed using chemoradiotherapy for those with positive margins and ENE. Radiotherapy was performed for patients with high-risk close margins and pathological node (pN) 2b, taking into account their general condition and acceptance [7,8].

Using the preoperative blood assessment results, the following inflammatory and nutritional markers were calculated: neutrophil-to-lymphocyte ratio (NLR); lymphocyte-to-monocyte ratio (LMR); platelet-to-lymphocyte ratio (PLR); prognostic nutritional index (PNI); monocyte-to-albumin ratio (MAR); and C-reactive protein-to-albumin ratio (CAR).

This study was conducted in accordance with the Declaration of Helsinki and was approved by the Institutional Review Board of the University of Tsukuba Hospital (No. R05-158). The requirement for informed consent was waived owing to the retrospective nature of the study.

### 2.2. Study Variables

SMI was identified as the primary predictive variable in this study. The primary outcome variables were overall survival (OS), disease-free survival (DFS), and SSI. The analysis also incorporated additional variables, including patient characteristics such as age, body mass index (BMI), clinical tumor classification, surgical margin, positive LN, pN classification, and ENE.

### 2.3. Data Analyses

Patients were stratified into two groups based on SMI cutoff values, and the χ^2^ test or Fisher’s exact probability test was used to compare the subgroups. Continuous variables in the subgroups were compared using the Mann–Whitney U test. Survival curves were obtained using the Kaplan–Meier method, and differences in OS and DFS were analyzed using the log-rank test. OS was calculated from the date of first diagnosis to death from any cause. DFS was calculated from the initial diagnosis until the development of any signs of cancer. The cutoff date for patients who had not yet succumbed to the disease was May 2024. Univariate and multivariate analyses of OS were performed using Cox proportional hazards models. All statistical analyses were performed using SPSS software version 29.0 for Macintosh (IBM Corp., Armonk, NY, USA). A *p*-value of less than 0.05 was deemed statistically significant.

## 3. Results

### 3.1. Patient Characteristics

Among the 92 patients with OSCC who underwent FFRS, 59 were males, 33 were females, and the average age was 66.0 (34–86) years. The median Eastern Cooperative Oncology Group performance status (PS) was 0 (range, 0–1) (Table 1).

The primary site and clinical TN classification are presented in Table 1. As many as 9 (9.9%) patients had clinical stage II, 11 (12.0%) had stage III, 58 (63.0%) had stage IVa, and 14 (15.2%) had stage IVb disease (Table 1). The free flap types performed were as follows: anterolateral thigh flap (ALT), 37 (40.2%) patients; fibula flap (FF), 31 (33.7%) patients; and rectus abdominis myocutaneous flap, 24 (26.1%) patients. SSI occurred in 11 (13.0%) patients, and wound dehiscence and delayed wound healing were observed in 22 (23.9%) patients. Five (5.4%) patients underwent reoperation owing to thrombosis, of whom three (3.3%) were successfully rescued. Seven patients (7.6%) developed partial necrosis, while two (2.2%) experienced complete necrosis. Systemic delirium occurred in 27 (29.3%) and postoperative pneumonia occurred in 21 (22.8%) patients. The pathological results of the primary site and neck are presented in Table 1 and Table 2, respectively. The median follow-up duration was 44.6 (range: 5.1–132.4) months. The prognosis included primary recurrence in 14 patients (15.2%), neck recurrence in 11 patients (12.0%), and distant metastasis in 20 patients (21.7%) (Table 1).

### 3.2. Clinical Characteristics of Patients Included in the Study Dichotomized by SMI Cutoff Value

The cutoff values determined using the median SMI values were 45.94 and 38.03 cm^2^/m^2^ for males and females, respectively. According to these cutoff values, the low and high SMI groups were dichotomized. As many as 47 (51.1%) patients had a low SMI, and 45 (48.9%) had a high SMI. BMI ≥ 18.5 kg/m^2^ was detected in 39 (83.0%) patients with a low SMI and 42 (93.3%) with a high SMI. A significant difference was detected between those aged <65 and ≥65 years (*p* = 0.037; Table 1). However, no significant difference in SMI was observed between patients with and without SSI or postoperative pneumonia. Additionally, no statistically significant differences were detected between SSI and the following variables: age, PS, BMI, presence of diabetes mellitus, albumin level, preoperative irradiation, or smoking. However, SSI rates were significantly different between patients who consumed alcohol and those who did not (*p* = 0.049).

For SMI, continuous variable analysis revealed a median bleeding volume of 374 mL (126–1031 mL) in the low SMI group and 510 mL (70–1278 mL) in the high SMI group, indicating a significant difference (*p* = 0.004). In the low SMI group, the duration of high care unit (HCU) and hospital stay was 7 (4–10) and 38 (22–152) days, respectively, whereas in the high SMI group, HCU and hospital stay duration were 7 (5–12) and 36 (19–98) days, respectively. No significant differences in inflammatory and nutritional markers were observed (Table 1).

### 3.3. Association Between Clinical Factors and OS and DFS

**Figure 2:**
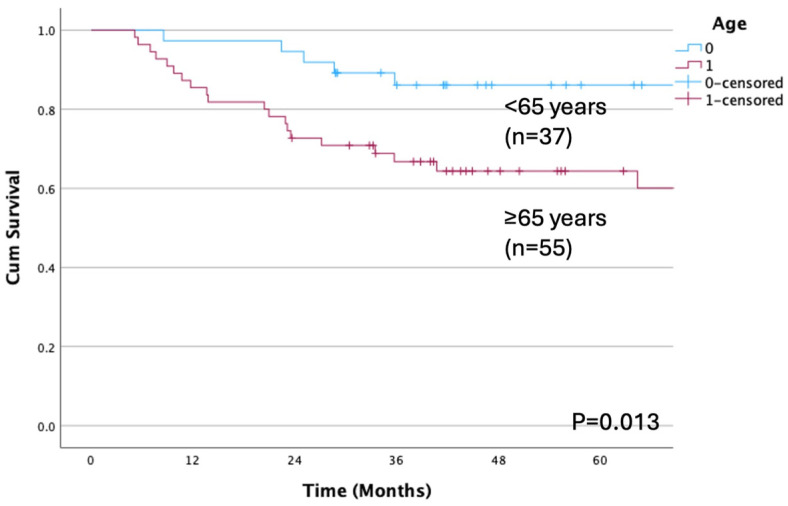
Overall survival rate according to age. A significant difference is observed between patients aged <65 years and ≥65 years (*p* = 0.013). The OS rates are 86.1% for those aged <65 years and 60.1% for those aged ≥65 years. OS, overall survival.

**Figure 3:**
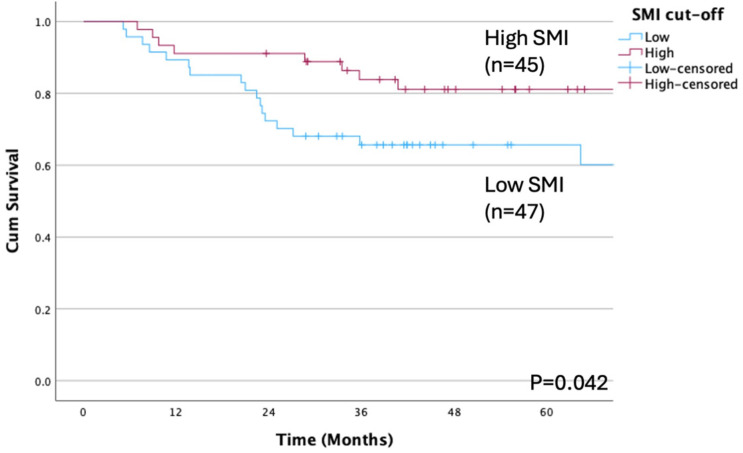
Overall survival rate according to SMI cutoff. A significant difference is observed between low and high SMI (*p* = 0.042). The OS rates are 60.2% and 81.1% in the low and high SMI groups, respectively. OS, overall survival; SMI, skeletal muscle index.

Table 2 presents the associations between the study variables and OS and DFS. Significant differences in OS were observed based on age and SMI classification. Patients aged ≥65 years had an OS of 60.1%, compared with 86.1% for those aged <65 years (*p* = 0.013; Figure 2). Similarly, patients with a high SMI had an OS of 81.1% and those with a low SMI had an OS of 60.2% (*p* = 0.042; Figure 3). Considering the primary site, OS differed significantly when patients were stratified by tongue, lower gingiva, and other sites (*p* < 0.001).

**Figure 4:**
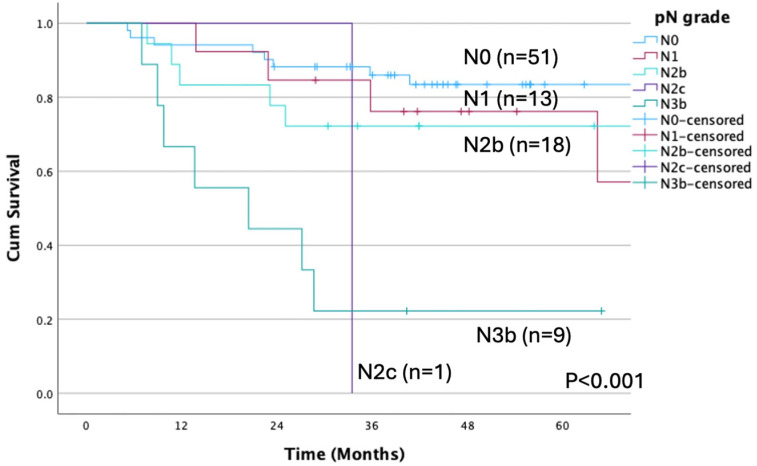
Overall survival rate according to pN grade (*p* < 0.001). A significant difference is observed between pN grades. The OS rate is 83.4% for N0, 57.1% for N1, 72.2% for N2b, 0% for N2c, and 22.2% for N3. pN, pathological node; OS, overall survival.

**Figure 5:**
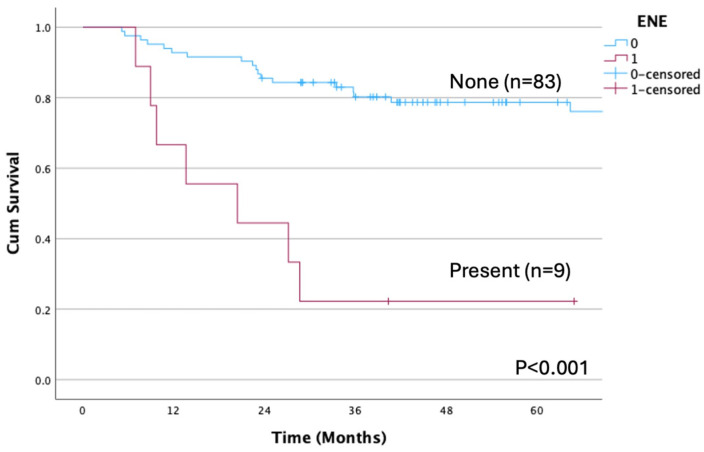
Overall survival according to ENE. A significant difference is observed between the ENE-present and ENE-none groups (*p* < 0.001). The OS rates are 22.2% and 76.1% for the ENE-present and ENE-none groups, respectively. ENE, extranodal extension; OS, overall survival.

**Figure 6:**
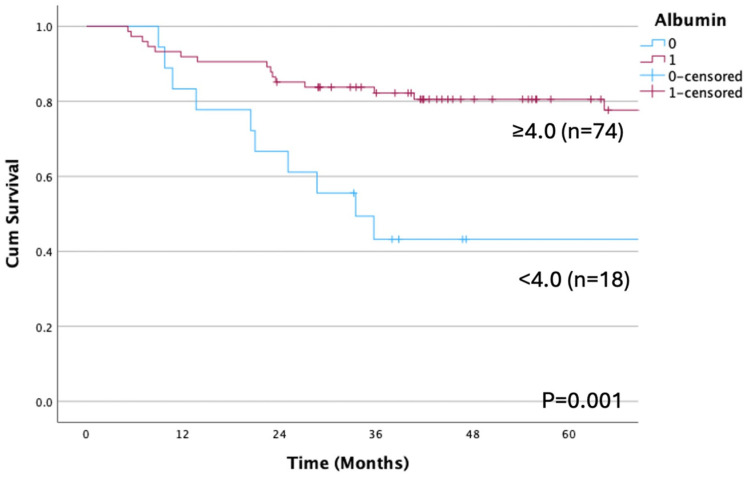
Overall survival rate according to albumin cutoff. A significant difference is observed between those with albumin levels <4.0 mg/dL and ≥4.0 mg/dL (*p* = 0.001). The OS rates are 43.2% and 77.7% in the low and high albumin groups. OS, overall survival.

OS differed significantly when patients were stratified according to pathological N status (*p* < 0.001; Figure 4). Moreover, OS rates differed significantly between N1-3b and N0 pN (*p* = 0.007), as well as between those with and without ENE (*p* < 0.001, Figure 5). Likewise, significant differences in OS were detected when patients were categorized by albumin <4.0 vs. ≥4.0 mg/dL (*p* = 0.001; Figure 6).

Considering the primary site, DFS differed significantly when patients were stratified by tongue, lower gingiva, and other sites (*p* < 0.001). The DFS differed significantly when patients were stratified according to pN classification (*p* < 0.001). Significant differences in DFS were observed for pN (N1-3b or N0) and ENE (*p* < 0.001). Similarly, significant differences in DFS were observed when patients were categorized by albumin <4.0 vs. ≥4.0 mg/dL (*p* = 0.018).

### 3.4. Cox Multivariate Regression Analysis

Univariate analyses revealed significant associations between OS and age (≥65 vs. <65 years; *p* = 0.019), SMI (low vs. high; *p* = 0.048), pN (present vs. none; *p* = 0.010), ENE (present vs. none; *p* < 0.001), and albumin (<4.0 vs. ≥4.0; *p* = 0.003). Cox multivariate analysis, which incorporated parameters identified through univariate and logistic multivariate analyses employing a stepwise forward selection approach, identified three independent predictive factors for OS: ENE (*p* = 0.004), SMI (*p* = 0.013), and albumin (*p* = 0.034). Twenty-five outcomes for OS, and two independent factors (ENE and SMI) were selected. The results included SMI (low vs. high; HR: 2.339, 95% CI: 1.008–5.429; *p* = 0.015) and ENE (present vs. absent; HR: 7.727, 95% CI: 3.083–19.368; *p* < 0.001) (Table 3).

Conversely, Cox multivariate analysis, utilizing parameters selected through univariate and logistic multivariate analyses with the stepwise forward selection method, identified independent variables and one independent predictive factor for DFS: pN (present vs. none; HR: 4.248, 95% CI: 1.813–9.953; *p* < 0.001) (Table 4).

## 4. Discussion

Recipient-site SSI reportedly occurs in approximately 25% of patients, and low SMI has been found to be a substantial risk factor for SSI [4,9]. In the current study, we detected no significant difference in SSI between the low and high SMI groups in patients undergoing FFRS for OSCC. Conversely, significant differences in OS were observed when patients were dichotomized by age, pN, ENE, and SMI. Cox multivariate analysis identified SMI and ENE as independent predictive factors for OS. Based on these findings, patients with a low SMI, as diagnosed through preoperative CT, need to improve their poor nutritional status to enhance the OS of OSCC.

An association between cancer prognosis and sarcopenia has been established [1,2,3]. Skeletal muscle loss in the postoperative acute phase may be a new predictor for long-term prognosis after highly invasive surgery, such as esophageal SCC surgery [10]. Patients with sarcopenia were shown to exhibit markedly poorer OS and DFS than those without sarcopenia [3]. Bonavolonta et al. reported that the presence of sarcopenia did not lead to a statistically significant reduction in OS in patients with OSCC; however, sarcopenic obesity exerted a notable negative prognostic impact [11]. Considering prognosis, statistically significant differences in OS, but not in DFS, have been reported between patients with low and high SMM [1]. In the current study, pN was identified as an independent predictor of DFS. In contrast with OS, DFS may be influenced by locoregional factors rather than systemic factors, such as sarcopenia.

Our results correspond with those reported previously, identifying a significant difference in OS but not DFS. A systematic review and meta-analysis of 10 studies explored the association of sarcopenia with oncologic outcomes of primary treatment among patients with OSCC, revealing that the cutoff value of L3 SMI was 36.02–52.4 cm^2^/m^2^ in males and 30.6–47.5 cm^2^/m^2^ in females. The percentage of patients with sarcopenia was 23–67% based on the cutoff value of stage I to IVB, excluding one article. The authors concluded that over one-third of patients with OSCC may present with sarcopenia and that pretreatment sarcopenia is associated with significantly worse OS and DFS [3]. In the current study, the SMI cutoff values were 45.94 and 38.03 cm^2^/m^2^ in males and females, respectively, for OS, which are within the range reported previously. Based on established cutoff values, the frequency of sarcopenia in patients with OSCC was found to be 16–67% in those with stage I to IVB disease, excluding one article [3,6]. In contrast, our study revealed that 51.1% of patients had sarcopenia, which was higher than reported in previous reviews. Patients who underwent FFRS and had a higher clinical stage were included; patients with sarcopenia were included because of dysfunction of oral intake.

A low SMM is reportedly a strong predictive factor for complications of FFRS and other postoperative complications in patients undergoing fibular free flap reconstruction [1]. Recipient-site SSI and lower SMI have been identified as notable risk factors for SSI [4,9]. Accordingly, enhancing SMM through exercise or nutrition before surgery may prevent recipient-site SSI after OSCC resection and subsequent FFRS. A meta-analysis assessing the association between low SMM and surgical complications was mainly performed using the Clavien–Dindo classification after FFRS in patients with head and neck cancer (HANC). Among the included studies, the prevalence of low SMM ranged from 24.6% to 61.5%. The meta-analysis revealed an OR of 2.42 (95% CI 1.53–3.32) for postoperative complications. Consequently, SMM was suggested as an independent risk factor for SSI and postoperative complications in patients undergoing HANC reconstructive surgery [6]. Conversely, the authors of a previous study investigated the predictive value of low SMM for postoperative complications in patients with T1–2 OSCC who had undergone neck dissection. The authors reported that a low SMM was not predictive of postoperative complications [12]. In our study, SSI occurred in only 12.0% of patients, and wound dehiscence and delayed wound healing were observed in 23.9% of patients. The SSI occurrence rate in our study was lower than that reported previously, and no significant difference in SSI was observed between the low and high SMI groups. Diagnostic criteria for recipient-site SSI within 30 days postoperatively were the same as those described previously. SSI was identified as purulent drainage from the incision, an incision that was deliberately opened or underwent spontaneous dehiscence due to infection, evidence of an abscess or deep infection on examination or imaging, or a diagnosis of an SSI by the surgeon [13]. Moreover, our study found no statistically significant differences between SSI and the following variables: age, PS, BMI, presence of diabetes mellitus, albumin level, and preoperative irradiation. These discrepancies may be attributed to the good general condition of patients undergoing FFRS in our study, superior surgical procedure, and prevention of SSI during the perioperative period.

SMM is typically assessed using abdominal CT at the L3 level. However, abdominal CT is not routinely included in preoperative management protocols for patients with OSCC and is often only available in a subset of patients with advanced disease and an increased risk of distant metastasis [1]. The C3 CSA was not precisely evaluated as it included metastatic lymph nodes and metal artifacts of the teeth, according to the head position. Lu et al. constructed a formula to predict the L3 skeletal muscle CSA from the C3 CSA 13. Regarding the data constructed from the formula, some authors have reported that C3 CSA data lack usefulness. Therefore, C3 SMM has not been reported as a strong predictor of L3 SMM in patients who have sarcopenia with HANC [14,15]. Conversely, Bril et al. reported that SMM measurements at the C3 level strongly correlated with those at L3. The assessment of SMM on head and neck CT is feasible and may be an alternative to abdominal CT imaging. This method allows the assessment of sarcopenia using routinely performed scans without additional imaging or patient burden [16,17].

In the present study, we evaluated the association between systemic inflammatory markers, such as NLR, LMR, PLR, PNI, MAR, CAR, and SMI. PLR and PNI showed significant differences between high and low SMI in the continuous variable analysis. In the multivariate analysis, sarcopenia showed a statistically significant association with poor OS, and high PLR ≥ 170.9 was an independent predictor for sarcopenia [18]. Our results revealed a median PLR of 149.54 and 123.43 in the low and high SMI groups, respectively, consistent with the findings of a previous report. Systemic inflammation is markedly associated with sarcopenia. A high PLR has been found to be associated with enhanced survival and oncological effects of sarcopenia. Thus, combining these two parameters is useful for identifying patients with OSCC at risk of poor survival outcomes.

The limitations of this study include the small number of available cases and the bias in patient selection for FFRS owing to the retrospective and single-institution design. In addition, multiple primary sites of oral cancer were included, and selecting cases involving the tongue or lower gingiva was prioritized. Accordingly, a prospective multi-center study is required to validate the preliminary findings of the present report with a larger sample size. Furthermore, although quality of life is a critical factor in oral cancer management, we did not determine whether patients with a low SMI could eat before surgery or their postoperative nutritional intake and oral function, considering its retrospective nature. In patients with HANC, exercise and nutrition interventions with progressive resistance training and oral nutritional supplements during and after radiotherapy were found to be feasible and effective strategies in mitigating muscle loss [19]. In gastrointestinal surgery, perioperative recommendations for malnutrition and sarcopenia have been reported as prognostic factors. In the case of high metabolic risk or severe malnutrition, surgery should be postponed, nutrition therapy should be administered enterally for 10–14 days, if possible, and prehabilitation should be considered. Preoperative use of oral nutrition for at least 7 days may reduce the rate of infectious complications and length of hospital stay. In patients with gastrointestinal cancer, preoperative immunonutrition for 5–7 days may reduce the incidence of infectious complications [20].

Accordingly, interventions designed to preserve muscle mass, such as multimodal preoperative rehabilitation programs incorporating physical therapy and nutritional intervention prior to surgery, could be effective in enhancing SMM and outcomes. In the future, developing a specific nutritional management program is imperative, given the temporal constraints that emerge prior to surgery.

## 5. Conclusions

In the study presented herein, SMI and ENE were identified as independent predictive factors for OS in patients with OSCC who underwent resection and FFRS. Screening for sarcopenia could be used as a tool to plan treatment strategies and monitor nutritional support during cancer treatment, including postoperative ENE management, potentially enhancing patient outcomes.

## Figures and Tables

**Table 1:** Clinical characteristics of patients included in the study dichotomized into low and high skeletal muscle mass index.

Variables		Total No. of Patients	Low SMI No. of Patients (%) *n* = 47	High SMI No. of Patients (%) *n* = 45	*p*-Value ^†^
Sex	Male	59	30 (63.8)	29 (64.4)	0.951
	Female	33	17 (36.2)	16 (35.6)	
Age (years)	<65	37	14 (29.8)	23 (51.1)	0.037 *
	≥65	55	33 (70.2)	22 (48.9)	
BMI (kg/m^2^)	<18.5	11	8 (17.0)	3 (6.7)	0.199
	≥18.5	81	39 (83.0)	42 (93.3)	
Tabaco consumption	Ever	45	25 (53.2)	20 (44.4)	0.401
	Never	47	22 (46.8)	25 (55.6)	
Alcohol consumption	Present	44	24 (51.1)	20 (44.4)	0.525
	Absent	48	23 (48.9)	25 (55.6)	
Primary site	Tongue	41	25 (53.2)	16 (35.6)	0.368
	Lower gingiva	35	15 (31.9)	20 (44.4)	
	Buccal mucosa	8	3 (6.4)	5 (11.1)	
	Floor of the mouth	7	4 (8.5)	3 (6.7)	
	Upper gingiva	1	0 (0)	1 (2.2)	
Clinical T classification	T2	17	9 (19.1)	8 (17.8)	0.722
	T3	15	9 (19.1)	6 (13.3)	
	T4a	47	24 (51.1)	23 (51.1)	
	T4b	13	5 (10.6)	8 (17.8)	
Clinical N classification	N0	41	25 (53.2)	16 (35.6)	0.140
	N1	11	3 (6.4)	8 (17.8)	
	N2b	35	15 (31.9)	20 (44.4)	
	N2c	4	3 (6.4)	1 (2.2)	
	N3b	1	1 (2.1)	0 (0)	
Stage classification	II	9	5 (10.6)	4 (8.9)	0.760
	III	11	7 (14.9)	4 (8.9)	
	IVa	58	29 (61.7)	29 (64.4)	
	IVb	14	6 (12.8)	8 (17.8)	
Histological grade	G1	49	21 (44.7)	28 (62.2)	0.122
	G2	34	19 (40.4)	15 (33.3)	
	G3	9	7 (14.9)	2 (4.4)	
Preoperative radiotherapy	Present	6	3 (6.4)	3 (6.7)	1.000
	Absent	86	44 (93.6)	42 (93.3)	
Pathological N classification	N0	51	27 (57.4)	24 (53.3)	0.657
	N1	13	7 (14.9)	6 (13.3)	
	N2b	18	10 (21.3)	8 (17.8)	
	N2c	1	0 (0)	1 (2.2)	
	N3b	9	3 (6.4)	6 (13.3)	
Surgical site infection	Present	11	5 (10.6)	6 (13.3)	0.690
	Absent	81	42 (89.4)	39 (86.7)	
Delirium	Present	27	15 (31.9)	12 (26.7)	0.581
	Absent	65	32 (68.1)	33 (73.3)	
Postoperative pneumonia	Present	21	8 (17.0)	13 (28.9)	0.175
	Absent	71	39 (83.0)	32 (71.1)	
Primary recurrence	Present	14	9 (19.1)	5 (11.1)	0.283
	Absent	78	38 (80.9)	40 (88.9)	
Neck recurrence	Present	11	8 (17.0)	3 (6.7)	0.199
	Absent	81	39 (83.0)	42 (93.3)	
Distant metastasis	Present	20	13 (27.7)	7 (15.6)	0.159
	Absent	72	34 (72.3)	38 (84.4)	
Free flap type	ALT	37	23 (48.9)	14 (31.1)	0.210
	FF	31	13 (27.7)	18 (40.0)	
	RAMF	24	11 (23.4)	13 (28.9)	
**Variables** **(Continuous)**			**Low SMI** **Median (Range)**	**High SMI** **Median (Range)**	***p*-Value ^††^**
HCU duration	(days)		7 (4–10)	7 (5–12)	0.411
Hospital stay duration	(days)		38 (22–152)	36 (19–98)	0.537
PS			0 (0–1)	0 (0–1)	0.692
ASA			2 (1–3)	2 (1–3)	0.540
Bleeding count	(mL)		374 (126–1031)	510 (70–1278)	0.004 **
Operative time	(h:min)		11:05 (8:24–15:15)	11:47 (8:20–15:31)	0.133
Albumin	(mg/dL)		4.2 (3.3–5.1)	4.2 (3.2–4.8)	0.820
NLR			2.74 (1.09–11.70)	2.39 (0.93–15.23)	0.246
LMR			4.38 (1.14–11.15)	5.43 (1.81–8.80)	0.060
PLR			149.54 (73.03–529.79)	134.81 (34.52–291.06)	0.193
PNI			116.90 (63.01–228.00)	127.45 (89.50–222.00)	0.119
MAR			88.64 (0–200.00)	90.24 (34.55–160.98)	0.919
CAR			0.0200 (0.0063–0.1921)	0.0136 (0.0063–0.6641)	0.379

^†^ Using Chi-square analysis or Fisher’s exact probability test. ^††^ Using Mann–Whitney’s U test. * *p* < 0.05, statistically significant difference. ** *p* < 0.01, statistically significant difference. SMI, skeletal muscle index; BMI, body mass index; T, tumor; N, node; RAMF, rectus abdominis myocutaneous flap; ALT, anterolateral thigh flap; FF, fibula flap; HCU, high care unit; PS, performance status; ASA, American Society of Anesthesiologists; NLR, neutrophil-to-lymphocyte ratio; LMR, lymphocyte-to-monocyte ratio; PLR, platelet-to-lymphocyte ratio; PNI, prognostic nutritional index; MAR, monocyte-to-albumin ratio; CAR, C-reactive protein-to-albumin ratio.

**Table 2:** Characteristics of patients with oral squamous cell carcinoma in relation to cumulative overall and disease-free survival.

Variables		No. of Patients (%)	OS (%)	*p* ^†^	DFS (%)	*p* ^†^
Age (years)	≥65	55 (59.8)	60.1	0.013 *	58.0	0.057
	<65	37 (40.2)	86.1		78.4	
Sex	Male	59 (64.1)	68.4	0.658	66.1	0.665
	Female	33 (35.9)	74.8		67.3	
BMI (kg/m^2^)	<18.5	11 (12.0)	54.5	0.051	54.5	0.231
	≥18.5	81 (88.0)	73.2		68.1	
SMI	High	45 (48.9)	81.1	0.042 *	81.4	0.070
	Low	47 (51.1)	60.2		65.4	
Tobacco consumption	Ever	45 (48.9)	74.3	0.535	64.4	0.601
	Never	47 (51.1)	68.4		68.7	
Alcohol consumption	Ever	44 (47.8)	76.3	0.442	68.2	0.804
	Never	48 (52.2)	66.5		65.2	
Diabetes mellitus	Present	21 (22.8)	71.4	0.929	66.7	0.965
	None	71 (77.2)	70.2		66.5	
Cardiovascular disease	Present	7 (7.6)	47.6	0.284	57.1	0.442
	None	85 (92.4)	73.3		67.3	
Cerebrovascular disease	Present	8 (8.7)	87.5	0.320	100	0.062
	None	84 (91.3)	69.0		63.3	
History of cancer	Present	16 (17.4)	80.2	0.400	80.8	0.241
	None	76 (82.6)	69.0		63.6	
Preoperative radiotherapy	Present	6 (6.5)	66.7	0.829	83.3	0.381
	Absent	86 (93.5)	70.8		65.3	
Primary site	Tongue	41 (44.6)	67.5	<0.001 **	63.4	<0.001 **
	Lower gingiva	35 (38.1)	100		100	
	Buccal mucosa	8 (8.7)	75.0		0	
	Floor of the mouth	7 (7.6)	0		42.9	
	Upper gingiva	1 (1.1)	100		100	
Clinical T classification	T2	17 (18.5)	74.9	0.871	64.7	0.904
	T3	15 (16.3)	80.0		64.0	
	T4a	47 (51.1)	67.8		68.0	
	T4b	13 (14.1)	68.4		61.5	
Clinical N classification	N0	41 (44.6)	77.4	0.014 *	78.0	0.018 *
	N1	11 (12.0)	77.9		53.0	
	N2b	35 (38.0)	70.4		65.7	
	N2c	4 (4.3)	25.0		25.0	
	N3b	1 (1.1)	0		0	
Clinical stage classification	II	9 (9.8)	77.8	0.803	66.7	0.830
	III	11 (12.0)	72.7		54.5	
	IVA	58 (63.0)	71.6		70.6	
	IVB	14 (15.2)	63.5		57.1	
Pathological surgical margin (mm)	≥5	51	73.3	0.059	69.0	0.076
	<5	34	73.5		67.6	
	Positive	5	0		20.0	
pN classification	N0	51 (55.4)	83.4	<0.001 **	86.2	<0.001 **
	N1	13 (14.1)	57.1		61.5	
	N2b	18 (19.6)	72.2		42.9	
	N2c	1 (1.1)	0		0	
	N3b	9 (9.8)	22.2		22.2	
pN	N1-3b	41 (44.6)	55.8	0.007 **	42.5	<0.001 **
	N0	51 (55.4)	83.4		86.2	
ENE	Present	9 (9.8)	22.2	<0.001 **	22.2	<0.001 **
	None	83 (90.2)	76.1		71.3	
Histological grade	G1	49 (53.3)	74.9	0.829	69.3	0.974
	G2	34 (37.0)	67.1		63.0	
	G3	9 (9.8)	66.7		66.7	
Albumin	<4.0	18 (19.6)	43.2	0.001 **	44.4	0.018 *
(mg/dL)	≥4.0	74(80.4)	77.7		72.0	

^†^ Using the log-rank test. * *p* < 0.05, statistically significant difference. ** *p* < 0.01, statistically significant difference. BMI, body mass index; SMI, skeletal muscle index; OS, overall survival; DFS, disease-free survival; T, tumor; N, node; pN, pathological node; ENE, extranodal extension.

**Table 3:** Univariate and multivariate Cox regression analyses for overall survival in the primary cohort.

	Univariate Analysis		Multivariate Analysis	
Variables	HR (95% CI)	*p*-Values ^†^	HR (95% CI)	*p*-Values ^†^
Age (years)	3.225 (1.209–8.602)	0.019 *		
≥65 vs. <65				
SMI				
Low vs. High	2.339 (1.008–5.429)	0.048 *	2.900(1.226–6.862)	0.015 **
pN				
N0 vs. N1-3b	3.008 (1.297–6.976)	0.010 *		
ENE				
Present vs. Absent	6.147 (2.527–14.949)	<0.001 **	7.727 (3.083–19.368)	<0.001 **
Albumin (mg/dL)				
<4.0 vs. ≥4.0	3.429 (1.532–7.676)	0.003 **		

^†^ Using Cox regression analyses. * *p* < 0.05, statistically significant difference. ** *p* < 0.01, statistically significant difference. BMI, body mass index; SMI, skeletal muscle index; pN, pathological node; ENE, extranodal extension; HR, hazard ratio; CI, confidence interval.

**Table 4:** Univariate and multivariate Cox regression analyses for disease-free survival in the primary cohort.

	Univariate Analysis		Multivariate Analysis	
Variables	HR (95% CI)	*p*-Values ^†^	HR (95% CI)	*p*-Value ^†^
pN				
N0 vs. N1-3b	4.445 (1.903–10.379)	<0.001 *	4.248 (1.813–9.953)	<0.001 **
ENE				
Present vs. Absent	3.384 (1.435–7.980)	0.005 **		
Albumin (mg/dL)				
<4.0 vs. ≥4.0	2.294 (1.069–4.921)	0.033 *	2.039 (0.944–4.406)	0.070

^†^ Using Cox regression analyses. * *p* < 0.05, statistically significant difference. ** *p* < 0.01, statistically significant difference. pN, pathological node; ENE, extranodal extension; HR, hazard ratio; CI, confidence interval.

## Data Availability

Data are unavailable due to privacy or ethical restrictions.
